# Evaluation of the Care Pathway in the Context of the Dispensing of Emicizumab (Hemlibra) in Community Pharmacies in France: Protocol for a Cross-sectional Study Based on the Kirkpatrick Model

**DOI:** 10.2196/43091

**Published:** 2023-03-08

**Authors:** Laurie Fraticelli, Julie Freyssenge, Emilie Promé-Combel, Eléonore Agnellet, Yesim Dargaud, Valérie Chamouard

**Affiliations:** 1 Laboratory P2S (Health Systemic Process), UR 4129 University Claude Bernard Lyon 1 University of Lyon Lyon France; 2 Research on Healthcare Performance RESHAPE, INSERM U1290 University Claude Bernard Lyon 1 University of Lyon Lyon France; 3 French Reference Center of Hemophilia and Rare Inherited Bleeding Disorder Louis Pradel Hospital, Groupement Hospitalier Est Hospices Civils de Lyon Bron France; 4 Pharmaceutical Unit Louis Pradel Hospital, Groupement Hospitalier Est Hospices Civils de Lyon Bron France

**Keywords:** hemophilia, care pathway, healthcare management, emicizumab, Kirkpatrick model, Kirkpatrick, emicizumab, dispense, pharmacy, pharmacist, pharmaceutic, dispensing, dispensary, dispensation, patient satisfaction, pharma, community pharma, community health, hospital pharma, prophylaxis, hemophilia, knowledge acquisition, learning, cross-sectional, e learn, e-learning, questionnaire, survey

## Abstract

**Background:**

Commercialized since 2019, emicizumab (Hemlibra) was available only in French hospital pharmacies for prophylaxis of hemophilia A with or without inhibitors. Since June 15, 2021, patients can choose between a hospital and community pharmacy. These changes in the care pathway have important organizational consequences for patients, their relatives, and health professionals. Two training programs are available for community pharmacists: the “HEMOPHAR” training program proposed by the national reference center for hemophilia and the Roche training program proposed by the laboratory that markets the product.

**Objective:**

The PASODOBLEDEMI study aims to evaluate the direct impact of the training programs provided to community pharmacists in the context of the dispensing of emicizumab, and to evaluate patients’ satisfaction with their treatment whether they choose dispensation from a community pharmacy or retained dispensation from the hospital pharmacy.

**Methods:**

We designed a cross-sectional study based on the 4-level Kirkpatrick evaluation model: the immediate reaction of community pharmacists following training (*Reaction*), the knowledge acquired during the training (*Learning*), the professional practice of community pharmacists during dispensing of the product (*Behavior*), and patients’ satisfaction related to the treatment whether it is dispensed from a hospital or from a community pharmacy (*Results*).

**Results:**

Considering that single outcome measures cannot adequately reflect the complexity of this new organization, the Kirkpatrick evaluation model provides 4 distinct outcomes: the immediate reaction after the HEMOPHAR training program, the level of knowledge acquired after the HEMOPHAR training program, the impact of training on professional practice, and patient satisfaction with access to emicizumab. We developed specialized questionnaires for each of the 4 levels of the Kirkpatrick evaluation model. All community pharmacists involved in dispensing emicizumab, whether they have followed the HEMOPHAR or the Roche training program or neither, were eligible for inclusion. All patients with severe hemophilia A were eligible, irrespective of inhibitor use, age, treatment with emicizumab, and whether they chose dispensation from a community pharmacy or retained dispensation from a hospital pharmacy.

**Conclusions:**

The new organization for dispensing emicizumab to patients with hemophilia A in French community pharmacies must be accompanied by optimal safety and quality conditions due to the risk of serious and urgent bleeding situations in the management of rare bleeding diseases. The elaboration of the PASODOBLEDEMI protocol has already a positive impact with the commitment of all health professionals, physicians, hospital and community pharmacists, and the patient community. The results will be disseminated among the French authorities and will enable, if necessary, proposing this access model to other rare diseases.

**Trial Registration:**

ClinicalTrials.gov NCT05449197, https://clinicaltrials.gov/ct2/show/NCT05449197?term=NCT05449197; ClinicalTrials.gov NCT05450640, https://clinicaltrials.gov/ct2/show/NCT05450640?term=NCT05450640

**International Registered Report Identifier (IRRID):**

DERR1-10.2196/43091

## Introduction

### Background

Severe hemophilia A is an inherited bleeding disorder due to the deficiency of clotting factor VIII (FVIII), which causes bleeds occurring internally into joints and muscles, or external bleeding from minor cuts, surgical procedures, or injuries. The frequency and intensity of bleeding depends on the level of FVIII deficiency. In high-income countries, the life expectancy of patients with severe hemophilia A is decreased by 37%, the prevalence is estimated to be approximately 6 per 100,000 males, and prevalence at birth is to be approximately 9.5 cases per 100,000 males [1]. In France, the prevalence of severe hemophilia A has been estimated to be approximately 2095 patients in September 2022 [2]. Conventional management consists of FVIII replacement therapy. To maintain a sufficient level of clotting factor in the bloodstream to prevent bleeds, patients with severe hemophilia A are typically prescribed prophylactic treatment that consists of regular administration of FVIII, for example, every day, week, or month, according to how long the factor lasts in the body. Although effective, this treatment can only be administered intravenously, and in France, as well as in other countries [3-6], dispensation FVIII is only available from hospital pharmacies; they both contribute to organizational and geographical constraints [7] as well as a significant mental burden for patients and their caregivers, particularly for the parents of children with hemophilia [8].

### Dispensing Circuit of Emicizumab in France

Emicizumab (Hemlibra) is a laboratory-engineered protein that works by performing a key function in the clotting cascade that is normally carried out by the FVIII protein [9-12]. It can be prescribed for routine prophylaxis to prevent or reduce the frequency of bleeding episodes in adults and children of all ages, newborn and older, with hemophilia A, with or without treatment with an FVIII inhibitor [13-15].

**Figure 1:**
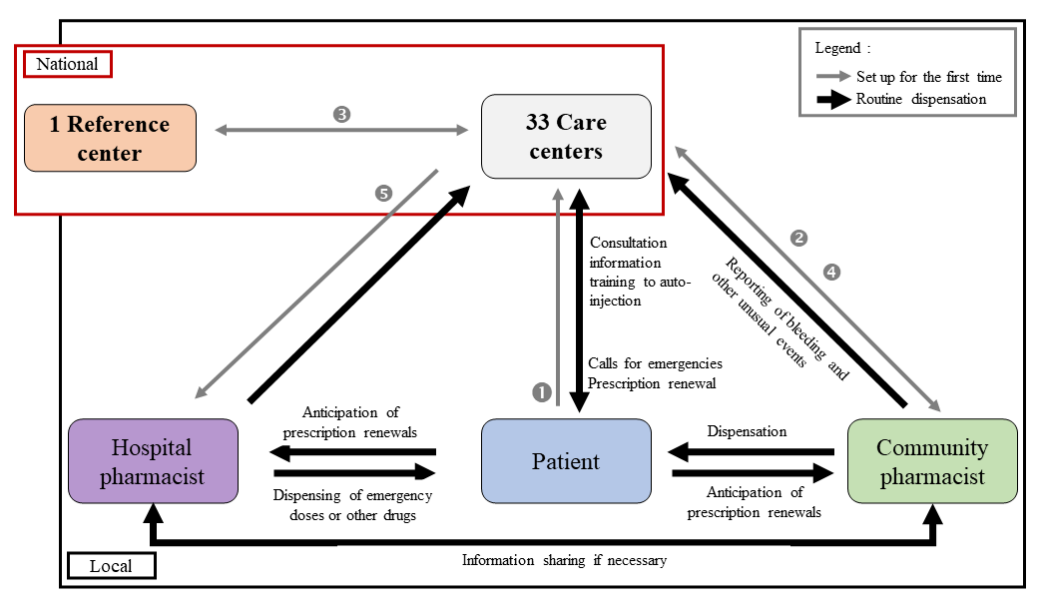
New organization of the dispensation circuit of emicizumab since June 15, 2021, in France. The black arrows indicate the routine dispensing organization, including emergency management of bleeding episodes. The gray arrows represent the setting up of the organization for the first time initiated by the patient, whose steps are numbered in the order of implementation. The patient indicates his/her chosen community pharmacy to one of the 33 care centers. The care center requests the connexion codes for the e-learning course "HEMOPHAR" from the reference center for hemophilia, and the community pharmacist is invited to follow the training. To facilitate the relationships between hospital and community pharmacists, practical information sheets are shared, including a contact sheet, checklist for dispensing emicizumab, and liaison sheet between the community pharmacist and the care center. The hospital pharmacy is informed by the care center about the transition of dispensing emicizumab in community pharmacy.

In 2019, emicizumab received marketing authorization for severe hemophilia A with or without an inhibitor in Europe [16]. This drug was initially available only from French hospital pharmacies, but from June 15, 2021, it has also been available in community pharmacies. The availability of emicizumab in community pharmacies was accompanied by the implementation of a rigorous new organization of the care pathway (Figure 1), ensuring that all professionals involved in the management of patients were identified by community pharmacists for an optimized exchange of information with the hospital pharmacy and the national reference center for hemophilia. Community pharmacists have the possibility to follow the training program by making a request to the reference center for hemophilia. The e-learning course called “HEMOPHAR” is based on the disease and its issues, and was designed by a multidisciplinary team of health care professionals from the reference center for hemophilia, as well as the platform for exchange and research on blood-derived medicines and their recombinant analogues (Plateforme d'Echange et de Recherche sur les Médicaments DErivés du Sang et leurs analogues recombinants) of the French society of clinical pharmacy (Société Française de Pharmacie Clinique). Also, a short training session organized by Roche, which that markets the drug, validated by the French medicines agency (Agence nationale de sécurité du médicament et des produits de santé) is proposed to pharmacists who order the drug; this is dispensed by telephone and aims to review the key points on the proper use of this medicinal product (including storage methods and ordering).

### Objectives of the PASODOBLEDEMI Study

These changes in the dispensing circuit have important organizational consequences for community pharmacists, becoming both new stakeholders and contacts in the patient care pathway [17]. The effectiveness of this new organization, and in particular the training, therefore needs to be evaluated. To address this, we designed a national study that aims to evaluate the direct impact of the training programs provided to community pharmacists in the context of the dispensing of emicizumab, and to evaluate patient satisfaction with their treatment whether they choose dispensation from a community pharmacy or retained dispensation from the hospital pharmacy.

## Methods

### Evaluation Strategy: the Kirkpatrick Model

#### Overview

The Kirkpatrick model (KM) represents a reference for the evaluation of training programs. Studies of various designs and purposes were addressed using the KM; for example, to evaluate a nutritional education program provided by health care professionals [18], to evaluate a train-the-trainer program for health care providers [19], to assess the training of cardiopulmonary resuscitation for nurses [20], to evaluate educational programs for undergraduate dental students regarding appropriate prescribing behavior during their early educational years [21], or to determine the effectiveness of a course for community health workers [22].

The popularity of the KM can be traced to several factors [23]. First, it addressed the need for training professionals to understand training evaluation in a systematic way by providing a straightforward language for communicating about training outcomes. Second, the KM provides trainers with a way to measure the results of what they do in terms of economic impacts and outcomes, which also measures the success of the organization. In addition, to simplify the complex process of training evaluation, a guide to the type of questions and the criteria that may be appropriate to focus on at different temporalities and information levels is provided.

#### Level 1: Reaction

The first level of evaluation consists of collecting immediate feedback from participants after completing the training. This information can be used to determine which elements of the training are working well or appreciated, and which ones need to be improved. Indicators such as participation rate, completion rate, or time spent on training are typically collected to supplement participant feedback.

#### Level 2: Learning

The second level is to accurately measure what participants did or did not learn during the training. Usually, the KM suggests a pre- and posttraining evaluation to quantify progress, for example, through knowledge tests or competency interviews. In contrast to immediate reactions to training, this level allows for the measurement of specific outcomes by identifying *Learning* objectives or targeting expected effects of training. When pretraining data are missing, the knowledge acquired post training is evaluated in accordance with the expected knowledge.

#### Level 3: Behavior

The third level is to determine if the training had a direct impact on the participants' behaviors. This level of evaluation is the most time-consuming because it takes several weeks or months for participants to build confidence and apply their knowledge in their professional practice. To measure *Behavior*, data collection and feedback experience are needed to give participants the opportunity to apply the skills they learned during training; for example, by exposing them to a simulated situation. It is important to mention that a lack of behavioral change does not necessarily mean that the training was ineffective.

#### Level 4: Results

The fourth level consists of assessing the impact of behavioral changes of the participants on the systemic organization, and in determining if the training reached the expected impact or improved the organization.

### Methodology

#### Study Design

**Figure 2:**
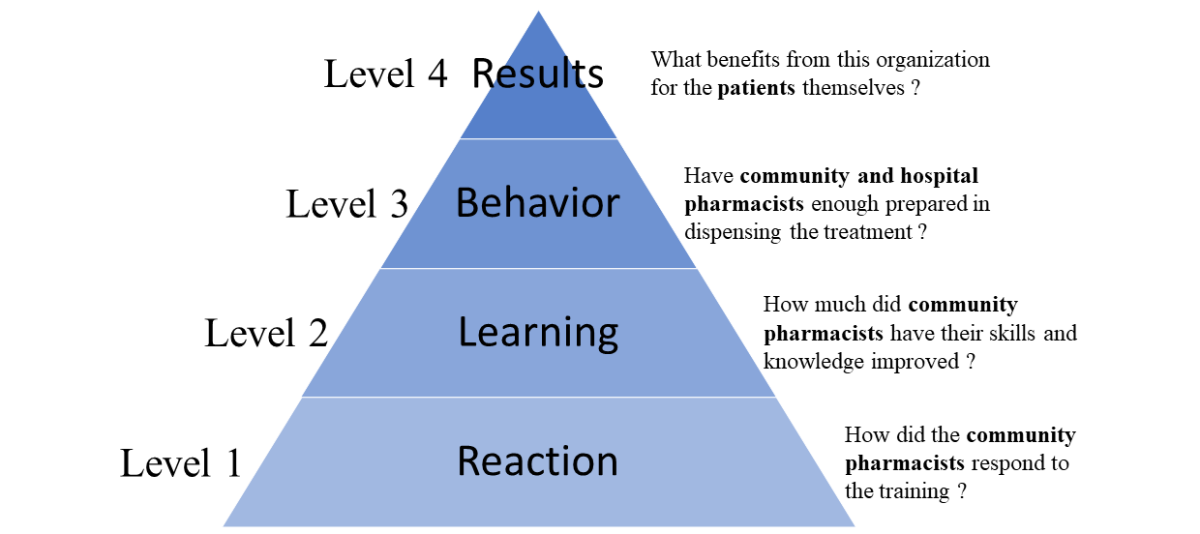
The Kirkpatrick model adapted to the PASODOBLEDEMI study.

We designed a cross-sectional study based on the KM. The first 3 levels are based on the data directly collected from the pharmacists. Level 1 refers to the evaluation of the immediate reaction of community pharmacists who followed the HEMOPHAR e-learning program and level 2 to their knowledge acquisition. Level 3 consists of evaluating the professional practice of both hospital and community pharmacists in the dispensation of emicizumab. The fourth level of the KM refers to the patients’ satisfaction related to treatment, whether dispensed from a hospital or a community pharmacy (Figure 2).

#### Study Populations

##### The Community Pharmacists Dispensing Emicizumab

Community pharmacists who received personalized login codes for the HEMOPHAR e-learning program from the national reference center for hemophilia were eligible for inclusion in this study for the evaluation of *Reaction* and *Learning*. All community pharmacists involved in the dispensing of emicizumab since June 15, 2021, whether they have followed the HEMOPHAR training program or the Roche training program or neither, were eligible for the evaluation of *Behavior*. Retired community pharmacists are excluded, as are those who have changed practice locations and are no longer practicing in a community pharmacy dispensing emicizumab.

##### Patients With Severe Hemophilia A With or Without an Inhibitor

All patients with severe hemophilia A, with or without an inhibitor, regardless of age, treatment with emicizumab, and whether they chose dispensation from a community pharmacy or retained dispensation from a hospital pharmacy, were eligible for the evaluation of the results. For those younger than 18 years, a legal representative fills out the questionnaire, and for those aged 18 years or older who need assistance in completing the questionnaire, this may be provided by caregivers or relatives.

#### Time Frames

A clinical research associate (CRA) is specifically dedicated to contact community pharmacists, informing them of the study modalities and outcomes, and inviting them to complete the e-questionnaires at the appropriate time (Table 1). Immediately after training is completed, the community pharmacists are invited to complete a questionnaire; this will be used to evaluate *Reaction*. The CRA is also able to incite community pharmacists—who have received their connexion codes but who have not yet started the HEMOPHAR learning program—to finalize the HEMOPHAR training program. During the HEMOPHAR training program, community pharmacists also complete 4 quizzes related to the following sections: presentation of the disease, therapeutic management, organization of care, and practice in the community pharmacy. The responses to the quizzes will be used to evaluate *Learning*. All community pharmacists are invited to complete the e-questionnaire related to *Behavior* at least 3 months after the implementation of the organization. Patients treated with emicizumab are invited to complete the appropriate questionnaire related to *Results*, an e-questionnaire specific to dispensation from hospital pharmacies, and a second e-questionnaire specific to dispensation from community pharmacies.

**Table 1:** Objectives, the eligible population, time frames, and measurement tool per levels of evaluation.

Levels	Objectives	Eligible population	Time frame	Measurement tool
Level 1: Reaction	To evaluate immediate reactions after the HEMOPHAR training program	Community pharmacists who followed the HEMOPHAR training program	Immediately after the HEMOPHAR training program	Short e-questionnaire and invitations sent by email
Level 2: Learning	To evaluate the level of knowledge acquired after the HEMOPHAR training program	Community pharmacists who followed the HEMOPHAR training program	During the HEMOPHAR training program	Responses to quizzes
Level 3: Behavior	To evaluate the impact of training on professional practice	All community pharmacists	At least 3 months after the implementation of the organization	e-Questionnaire and invitations sent by email
Level 4: Results	To evaluate the patients’ satisfaction regarding access to emicizumab	All patients treated with emicizumab	At least 3 months after the implementation of the organization	e-Questionnaire available with a QR code or specific URL

#### Development of the Questionnaires

The e-questionnaire related to *Reaction* was intentionally short and composed of 8 questions, including 5 questions based on the 4-item Likert-scale (not at all satisfied, not very satisfied, somewhat satisfied, and very satisfied) and 3 closed-ended questions (yes/no; Multimedia Appendix 1). The estimated time to complete this e-questionnaire is estimated to be approximately 1 minute.

The e-questionnaire related to *Behavior* was composed of 13 multiple-choice questions (Multimedia Appendix 2). The estimated time to complete this questionnaire is estimated to be approximately 8 minutes.

The e-questionnaires related to *Results* were adapted to the dispensing choice of the patient (Multimedia Appendices 3 and 4). They were composed of 33 questions. The estimated time to complete this questionnaire is estimated to be approximately 6 minutes.

The content of the questionnaires was initially defined by a multidisciplinary team composed of hospital and community pharmacists and research scientists specialized in methodology and care accessibility. A scientific committee was specifically constituted for the PASODOBLEDEMI study in order to challenge and validate the purposes, methodology, questionnaires, and expected outcomes. This committee was coordinated by the reference center for hemophilia and composed of hospital and community pharmacists, physicians, and the French association of patients with hemophilia (Association Française des Hémophiles). Following the recommendations of the scientific committee, the content of the e-questionnaires has evolved by integrating operational and organizational aspects and optimizing literacy. The e-questionnaires were finally tested in real-life conditions on a sample of eligible populations. The content of the questionnaires was finally validated by the scientific committee in July 2022.

#### Ethical Considerations

The study complies with the reference methodology for studies and evaluations in the health field [24]; therefore, it did not require written consent to participate. The ethics committee of the Hospices Civils de Lyon approved the study (n°2022-06-01 obtained on June 14, 2022). The PASODOBLEDEMI study was registered in ClinicalTrials.gov (NCT05449197 related to the evaluation of *Reaction*, *Learning*, and *Behavior*, and NCT05450640 related to the evaluation of *Results*).

## Results

### Administration

All e-questionnaires may be filled out using a PC or mobile device. The hospital and community pharmacists are invited by email to complete the e-questionnaires accessible using a login and password sent by the CRA for the evaluation of *Reaction* and *Behavior*; this also allowed the linking of data between evaluation levels. The e-questionnaire related to *Results* is accessible to eligible patients via a QR code or a specific URL presented on a paper information note, hand-delivered by the community pharmacist or hospital pharmacist when the patient comes for treatment or consultation.

### Data Collection

Data collection for the first 3 levels started on July 1, 2022, and for the fourth level, the questionnaires for patients were available since September 12, 2022. The study ended on December 31, 2022.

## Discussion

### Anticipated Findings

The availability of emicizumab in community pharmacies represents a significant advancement of the care pathway for patients with hemophilia A. The direct expected benefit concerns the improvement of the patients’ satisfaction by reducing the mental burden due to the geographical and organizational constraints to hospital pharmacies [8]. As observed in the management of other diseases, distance to the pharmacy may play a role in pharmaceutical management, leading, for example, to inappropriate and inadequate medication and management of asthma for patients living further from their preferred pharmacy [25]. Also, the dispensing by community pharmacies may provide better access and satisfaction to the patients [26]. For example, it is reported that the dispensing in community pharmacies of medicines previously only available from hospital pharmacies during the first wave of the COVID-19 pandemic in Portugal led to a significant increase in satisfaction levels in all evaluated domains: pharmacist’s availability, opening hours, waiting time, privacy conditions, and overall experience [27]. In the United States, satisfaction of patients with schizophrenia was improved by dispensing a long-acting injectable medication in community pharmacies during the COVID-19 pandemic [28]. In addition, a collaboration between opioid treatment programs and community pharmacists supported the feasibility and acceptability of physician prescribing and community pharmacy for access to methadone [29].

The new organization for dispensing emicizumab to patients with hemophilia A in French community pharmacies must be accompanied by optimal safety and quality conditions due to the risk of serious and urgent bleeding situations in the management of rare bleeding diseases. Based on the KM, the PASODOBLEDEMI study focuses on the evaluation of the training program and patient satisfaction 1 year after the implementation of the community-based dispensing of emicizumab. Widely used in the literature [30], the KM presents advantages, including ease of implementation and provision of evaluation of the systemic impacts on the organization. Considering that single outcome measures cannot adequately reflect the complexity of the organization, the KM provides 4 distinct outcomes.

The KM presents limitations [23] because the 4-level implementation can be time-consuming and requires an expensive investment to collect data and analyze learning outcomes. It is also difficult to establish a direct link between outcomes and training.

Other methodologies have been developed and used for evaluation of training programs, but which were not adapted to the PASODOBLEDEMI study. For instance, the Context-Inputs-Process-Product model (or the Stufflebeam model) [31,32] is based on the comparison of pre- and posttraining program evaluation to assess the gain in knowledge and confidence of participants; the Levels of Learning model (or the Kaufman model) is based on the use of specific evaluation scales (Kaufman Test of Educational Achievement) as an individually administered battery that provides in-depth assessment and evaluation of skills [33], but the design of this model is too rigid for the objectives of this study; and the Success Case Method (or the Brinkerhoff model) that is closer to an economically oriented performance scale with a study design based on a survey and semistructured interviews [34].

### Conclusions

The elaboration of the PASODOBLEDEMI protocol has already had a positive impact with the commitment of all health professionals, physicians, hospital and community pharmacists, as well as the patient community. The results will be disseminated to the French authorities and will enable, if necessary, proposing this access model to other rare diseases.

## Appendix

Multimedia Appendix 1 Questionnaire for the evaluation of Reaction.

Multimedia Appendix 2 Questionnaire for the evaluation of Behavior.

Multimedia Appendix 3 Questionnaire for the evaluation of Results (To patients or legal representatives or caregivers who have chosen community pharmacy dispensing).

Multimedia Appendix 4 Questionnaire for the evaluation of Results (To patients or legal representatives or caregivers who have retained hospital pharmacy dispensing).

## Abbreviations

CRA: clinical research associate
FVIII: clotting factor VIII
KM: Kirkpatrick model

## Data Availability

The data sets generated during and/or analyzed during the current study are not publicly available due to their confidentiality but aggregated results are available from the corresponding author on reasonable request.

## References

[1] Iorio A, Stonebraker JS, Chambost H, Makris M, Coffin D, Herr C, Germini F, DataDemographics Committee of the World Federation of Hemophilia (2019). Establishing the prevalence and prevalence at birth of hemophilia in males: a meta-analytic approach using national registries. Ann Intern Med.

[2] Données démographiques : Hémophilie A. Hôpitaux de Marseille.

[3] MASAC Document 188 - Recommendations Regarding Standards of Service for Pharmacy Providers of Clotting Factor Concentrates for Home Use to Patients with Bleeding Disorders. National Hemophilia Foundation.

[4] Malouin RA, Mckernan L, Forsberg A, Cheng D, Drake J, McLaughlin K, Trujillo M (2018). Impact of the 340B Pharmacy Program on services and supports for persons served by hemophilia treatment centers in the United States. Matern Child Health J.

[5] Valentino LA, Baker JR, Butler R, Escobar M, Frick N, Karp S, Koulianos K, Lattimore S, Nugent D, Pugliese JN, Recht M, Reding MT, Rice M, Thibodeaux CB, Skinner M (2021). Integrated hemophilia patient care via a national network of care centers in the United States: a model for rare coagulation disorders. J Blood Med.

[6] Noone D, O'Mahony B, Peyvandi F, Makris M, Bok A (2020). Evolution of haemophilia care in Europe: 10 years of the principles of care. Orphanet J Rare Dis.

[7] Leroy V, Freyssenge J, Renard F, Tazarourte K, Négrier Claude, Chamouard V (2019). Access to treatment among persons with hemophilia: A spatial analysis assessment in the Rhone-Alpes region, France. J Am Pharm Assoc (2003).

[8] Chamouard V, Fraticelli L, Freyssenge J, Claustre C, Négrier Claude, El Khoury C, PHAREO investigators (2022). PHAREO study: Perceived and observed accessibility to therapeutic drugs used for treating patients with inherited bleeding disorders. J Clin Pharm Ther.

[9] Yang R, Wang S, Wang X, Sun J, Chuansumrit A, Zhou J, Schmitt C, Hsu W, Xu J, Li L, Chang T, Zhao X (2022). Prophylactic emicizumab for hemophilia A in the Asia-Pacific region: A randomized study (HAVEN 5). Res Pract Thromb Haemost.

[10] Oldenburg J, Mahlangu JN, Bujan W, Trask P, Callaghan MU, Young G, Asikanius E, Peyvandi F, Santagostino E, Kruse-Jarres R, Negrier C, Kessler C, Xu J, Windyga J, Shima M, von Mackensen S (2019). The effect of emicizumab prophylaxis on health-related outcomes in persons with haemophilia A with inhibitors: HAVEN 1 Study. Haemophilia.

[11] Pipe SW, Shima M, Lehle M, Shapiro A, Chebon S, Fukutake K, Key NS, Portron A, Schmitt C, Podolak-Dawidziak M, Selak Bienz N, Hermans C, Campinha-Bacote A, Kiialainen A, Peerlinck K, Levy GG, Jiménez-Yuste Victor (2019). Efficacy, safety, and pharmacokinetics of emicizumab prophylaxis given every 4 weeks in people with haemophilia A (HAVEN 4): a multicentre, open-label, non-randomised phase 3 study. Lancet Haematol.

[12] Skinner MW, Négrier Claude, Paz-Priel I, Chebon S, Jiménez-Yuste Victor, Callaghan MU, Lehle M, Niggli M, Mahlangu J, Shapiro A, Shima M, Campinha-Bacote A, Levy GG, Oldenburg J, von Mackensen S, Pipe SW (2021). The effect of emicizumab prophylaxis on long-term, self-reported physical health in persons with haemophilia A without factor VIII inhibitors in the HAVEN 3 and HAVEN 4 studies. Haemophilia.

[13] Sharathkumar A, Lillicrap D, Blanchette VS, Kern M, Leggo J, Stain AM, Brooker L, Carcao MD (2003). Intensive exposure to factor VIII is a risk factor for inhibitor development in mild hemophilia A. J Thromb Haemost.

[14] Kempton CL, White GC (2009). How we treat a hemophilia A patient with a factor VIII inhibitor. Blood.

[15] Xi M, Makris M, Marcucci M, Santagostino E, Mannucci PM, Iorio A (2013). Inhibitor development in previously treated hemophilia A patients: a systematic review, meta-analysis, and meta-regression. J Thromb Haemost.

[16] Susen S, Gruel Y, Godier A, Harroche A, Chambost H, Lasne D, Rauch A, Roullet S, Fontana P, Goudemand J, de Maistre E, Chamouard V, Wibaut B, Albaladejo P, Négrier Claude (2019). Management of bleeding and invasive procedures in haemophilia A patients with inhibitor treated with emicizumab (Hemlibra ): Proposals from the French network on inherited bleeding disorders (MHEMO), the French Reference Centre on Haemophilia, in collaboration with the French Working Group on Perioperative Haemostasis (GIHP). Haemophilia.

[17] Beny K, du Sartz de Vigneulles B, Carrouel F, Bourgeois D, Gay V, Negrier C, Dussart C (2022). Haemophilia in France: modelisation of the clinical pathway for patients. Int J Environ Res Public Health.

[18] Zielińska-Tomczak Ł, Przymuszała P, Tomczak S, Krzyśko-Pieczka I, Marciniak R, Cerbin-Koczorowska M (2021). How Do Dieticians on Instagram Teach? The Potential of the Kirkpatrick Model in the Evaluation of the Effectiveness of Nutritional Education in Social Media. Nutrients.

[19] Kienlin S, Poitras M, Stacey D, Nytrøen Kari, Kasper J (2021). Ready for SDM: evaluating a train-the-trainer program to facilitate implementation of SDM training in Norway. BMC Med Inform Decis Mak.

[20] Dorri S, Akbari M, Dorri Sedeh M (2016). Kirkpatrick evaluation model for in-service training on cardiopulmonary resuscitation. Iran J Nurs Midwifery Res.

[21] Badran AS, Keraa K, Farghaly MM (2022). Applying the Kirkpatrick model to evaluate dental students' experience of learning about antibiotics use and resistance. Eur J Dent Educ.

[22] Firooznia M, Hamta A, Shakerian S (2020). The effectiveness of in-service training "pharmacopeia home health" based on Kirkpatrick's model: A quasi-experimental study. J Educ Health Promot.

[23] Bates R (2004). A critical analysis of evaluation practice: the Kirkpatrick model and the principle of beneficence. Eval Program Plann.

[24] Méthodologie de référence MR-004. Commission Nationale de l'Informatique et des Libertés.

[25] Deshpande M, Zahnd WE, Bandy L, Lorenson J, Fifer A (2019). Spatial analysis of disparities in asthma treatment among adult asthmatics. Res Social Adm Pharm.

[26] Pizetta B, Raggi LG, Rocha KSS, Cerqueira-Santos S, de Lyra-Jr DP, Dos Santos Júnior Genival Araujo (2021). Does drug dispensing improve the health outcomes of patients attending community pharmacies? A systematic review. BMC Health Serv Res.

[27] Murteira R, Romano S, Teixeira I, Bulhosa C, Sousa S, Conceição Maria Inês, Fonseca-Silva A, Martins H, Teixeira Rodrigues A (2022). Real-world impact of transferring the dispensing of hospital-only medicines to community pharmacies during the COVID-19 pandemic. Value Health.

[28] Mascari LN, Gatewood SS, Kaefer TN, Nadpara P, Goode JR, Crouse E (2022). Evaluation of patient satisfaction and perceptions of a long-acting injectable antipsychotic medication administration service in a community-based pharmacy during the COVID-19 pandemic. J Am Pharm Assoc (2003).

[29] Brooner RK, Stoller KB, Patel P, Wu L, Yan H, Kidorf M (2022). Opioid treatment program prescribing of methadone with community pharmacy dispensing: pilot study of feasibility and acceptability. Drug Alcohol Depend Rep.

[30] Newstrom JW (1995). Evaluating training programs: The four levels, by Donald L. Kirkpatrick. (1994). San Francisco: Berrett-Koehler. 229 pp., $32.95 cloth. Hum Resour Dev Q.

[31] Kang J, Park K (2017). Development of evaluation indicators for hospice and palliative care professionals training programs in Korea. J Contin Educ Health Prof.

[32] McMahon M, LaRocco S (2021). An international collaboration: linguistic editing of scholarly work. J Prof Nurs.

[33] Vidovic JL, Cornell MC, Frampton SE, Shillingsburg MA (2021). Adventures in direct instruction implementation: the devil is in the details. Behav Anal Pract.

[34] Medina L, Acosta-Pérez E, Velez C, Martínez G, Rivera M, Sardiñas L, Pattatucci A (2015). Training and capacity building evaluation: maximizing resources and results with Success Case Method. Eval Program Plann.

