# Supplementary material for: Evaluation of the Care Pathway in the Context of the Dispensing of Emicizumab (Hemlibra) in Community Pharmacies in France: Protocol for a Cross-sectional Study Based on the Kirkpatrick Model
Source: JMIR Res Protoc. 2023 Mar 8;12:e43091. doi: 10.2196/43091 (PMC10034610; doi:10.2196/43091)
Supplement: Multimedia Appendix 1 [file resprot_v12i1e43091_app1.docx]

**Multimedia Appendix 1**: Questionnaire for the evaluation of Reaction

The objective of this short questionnaire is to collect your satisfaction with the training.

Estimated time: 1 minute

Date: _ _ _ _ / _ _ / _ _

1. What is your overall level of satisfaction?

◻ Not at all satisfied ◻ Not very satisfied ◻ Somewhat satisfied ◻ Very satisfied

1. Does the content of the training session correspond to your expectations?

◻ Not at all satisfied ◻ Not very satisfied ◻ Somewhat satisfied ◻ Very satisfied

1. What is your level of satisfaction with the communication format?

◻ Not at all satisfied ◻ Not very satisfied ◻ Somewhat satisfied ◻ Very satisfied

1. Do you consider the training relevant to your professional practice?

◻ Not at all relevant ◻ Not very relevant ◻ Somewhat relevant ◻ Very relevant

1. What is your level of satisfaction with the duration of training?

◻ Not at all satisfied ◻ Not very satisfied ◻ Somewhat satisfied ◻ Very satisfied

1. Would you recommend the training session to your colleagues?

◻ Yes ◻ No

1. Have you ever taken any other training session?

◻ Yes, which one: ___________ ◻ No

1. Do you plan to take any other training session in the future?

◻ Yes, which one: ___________ ◻ No
