# Supplementary material for: Evaluation of the Care Pathway in the Context of the Dispensing of Emicizumab (Hemlibra) in Community Pharmacies in France: Protocol for a Cross-sectional Study Based on the Kirkpatrick Model
Source: JMIR Res Protoc. 2023 Mar 8;12:e43091. doi: 10.2196/43091 (PMC10034610; doi:10.2196/43091)
Supplement: Multimedia Appendix 2 [file resprot_v12i1e43091_app2.docx]

**Multimedia Appendix 2**: Questionnaire for the evaluation of Behavior

The objective of this questionnaire is to collect the frequency of situations encountered, the presence of possible difficulties during your professional practice in order to reinforce the support system for the dispensing of emicizumab in pharmacies.

Estimated time: 8 minutes

Date: _ _ _ _ / _ _ / _ _

**About the dispensation of emicizumab (Hemlibra ®) in your community pharmacy**

1. Number of hemophilia A patients who have chosen your community pharmacy: _ _ patients
2. Have you personally dispensed emicizumab (Hemlibra®)? ◻ yes ◻ no

If yes,

- Do you have the following documents?

Liaison sheet ◻ yes ◻ no

Contact information of the interveners ◻ yes ◻ no

Check-list ◻ yes ◻ no

- Do you feel that you had enough information? ◻ yes ◻ no

1. Have you ever been trained to dispense emicizumab?

◻ yes : ◻ HEMOPHAR ◻ Roche

- How many months have passed since the last training? _ _ months

◻ No, I have not taken any training session

**About your usual relationship with the patient**

1. Do you discuss the following concepts with the patient during the dispensing process?

|  | Never | Sometimes | Often | Every time |
| --- | --- | --- | --- | --- |
| Mode of action/ indication for emicizumab | ◻ | ◻ | ◻ | ◻ |
| How to administer the drug | ◻ | ◻ | ◻ | ◻ |
| How to use the administration kit | ◻ | ◻ | ◻ | ◻ |
| Importance of medication adherence/compliance | ◻ | ◻ | ◻ | ◻ |
| Management in the event of a missed dose | ◻ | ◻ | ◻ | ◻ |
| Impact of drug interactions, in case of self-medication | ◻ | ◻ | ◻ | ◻ |
| Reminder of what to do in case of a bleeding emergency according to medical recommendations/prescriptions | ◻ | ◻ | ◻ | ◻ |
| Reminder of the possible use of emergency doses of FVIII or agents by-passant according to the prescription | ◻ | ◻ | ◻ | ◻ |
| Management of possible side effects and their reporting to pharmacovigilance | ◻ | ◻ | ◻ | ◻ |

1. Have you been asked to provide pharmaceutical advice for the management of a minor bleeding episode? ◻ yes ◻ no

- If yes, did you recommend the use of:

◻ Arnica ◻ Ice ◻ Hemostatic ointment ◻ Other

1. Do you need a therapeutic education support, such as a brochure or other material, to facilitate your contact with the patient? ◻ yes ◻ no

**About the emergency management**

1. Have you ever been confronted with the management of an emergency situation (epistaxis, other bleeding episodes, fall...)? ◻ yes ◻ no

- If so, what actions have you taken?

◻ Referral to the Hemophilia Treatment Center

◻ Referral of the patient to the nearest hospital pharmacy

◻ Sharing information with another health professional:

◻ Physician / Nurse of the Hemophilia Treatment Center

◻ Hospital pharmacist

◻ Other, specify: ……………………………..

**About possible difficulties encountered during dispensing**

1. Did you use a third party to manage the situation?

◻ yes, specify: ◻ physician of the Hemophilia Treatment Center ◻ Hospital pharmacist

◻ colleague pharmacist of the community pharmacy ◻ other: _______

◻ no

1. Have you encountered any difficulties in the continuity of the patient's care?

◻ yes, specify: ◻ When a pharmacist on your team is on vacation

◻ If the patient changes pharmacies

◻ Other, specify: ……………………………..

◻ no

**About the organization of your community pharmacy**

1. Does the patient anticipate the refill of his or her prescription before coming to the hospital?

◻ at each dispensation ◻ often ◻ sometimes ◻ never

1. Does the prescription explicitly mention the dosage of the vials to be ordered?

◻ each time ◻ often ◻ sometimes ◻ never

1. What is the average time it takes for your wholesaler to supply Hemlibra®? _ _ days
2. Have you encountered any supply problems from the wholesale distributor?

◻ each time ◻ often ◻ sometimes ◻ never

1. Is your refrigerated storage cabinet equipped with an alarm? ◻ yes ◻ no
2. Have you encountered any problems with storage outside the refrigerated enclosure?

◻ each time ◻ often ◻ sometimes ◻ never

1. Is the pharmacy team - other than the pharmacist - involved in dispensing? ◻ yes ◻ no

- If yes,
- Which member of the pharmaceutical team is involved?

◻ Pharmacy assistant ◻ Pharmacy student

- How were these staff trained in dispensing?

◻ By a trained pharmacist ◻ By online training ◻ No training ◻ Other: ………………

- How do you control this dispensation and the advice of good use?

◻ By a trained pharmacist ◻ By an online training ◻ Other: …………………

1. If necessary, do you have a confidentiality area in the pharmacy where you practice? ◻ yes ◻ no

- If yes, do you use it for emicizumab dispensing?

◻ at the 1st dispensation ◻ at every dispensation ◻ often ◻ sometimes ◻ never ◻ other:………
