# Supplementary material for: Evaluation of the Care Pathway in the Context of the Dispensing of Emicizumab (Hemlibra) in Community Pharmacies in France: Protocol for a Cross-sectional Study Based on the Kirkpatrick Model
Source: JMIR Res Protoc. 2023 Mar 8;12:e43091. doi: 10.2196/43091 (PMC10034610; doi:10.2196/43091)
Supplement: Multimedia Appendix 3 [file resprot_v12i1e43091_app3.docx]

**Multimedia Appendix 3 :** Questionnaire for the evaluation of Results (To patients or legal representatives or caregivers who have chosen community pharmacy dispensing)

The purpose of this study is to evaluate the level of satisfaction of people with hemophilia, or their legal representative in the case of minors, or their caregiver/relative in the case of people with hemophilia, who have chosen to continue dispensing emicizumab (Hemlibra®) treatment in a **community pharmacy**.

Estimated time: 6 minutes

Date : _ _ _ _ /_ _ /_ _

**About you**

I am:

◻ The person with hemophilia

◻ The legal representative of a minor with hemophilia

◻ The caregiver / relative of the person with hemophilia

**About your household**

How many people live in your household? _ _ including _ _ minors (< 18 years old)

How many people have hemophilia? _ _ including _ _ minors (< 18 years old)

How many people are affected by emicizumab (Hemlibra®) treatment?

_ _ including _ _ minors (< 18 years old)

**About the patient and his pathology**

Sex of the patient: ◻ Female ◻ Male

Age of the patient: ◻ 0-4 years ◻ 5-10 years ◻ 11-14 years ◻ 15-17 years ◻ 18-30 years ◻ 31-40 years ◻ 41-50 years ◻ 51-60 years ◻ 61-70 years ◻ 71-80 years ◻ + 81 years

Weight: _ _ _ Kgs

Haemophilia A:

◻ With inhibitor ◻ Without inhibitor Factor VIII level: _ _ % ◻ do not know

Severity: ◻ minor (5 à <40%) ◻ moderate (1 à <5%) ◻ major (<1%)

At what age was your hemophilia diagnosed:

◻ at birth ◻ during childhood (1 to 12 years) ◻ in adolescence (13 to 17 years old) ◻ in adulthood (>18 years)

Handicap recognized by the *Maison Départementale des Personnes Handicapées*:

◻ yes: _ _ % invalidity ◻ no ◻ do not know

**About the choice of emicizumab (Hemlibra®) treatment**

Emicizumab dispensed for (month/year): _ _ /_ _ _ _

Emicizumab dispensed in community pharmacy for (month/year): _ _ /_ _ _ _

Your dose per injection is equal to: _ _ _ mg or _ _ _ ml or ◻ do not know

Your injection rhythm is as follows:

◻ 1 time per week ◻ 1 time every 14 days ◻ 1 time per month

You use the following vials (several choices possible depending on the dosage and color of the vials): ◻ ● 30mg/1mL ◻ ● 60mg/0.4mL ◻ ● 105 mg/0.7mL ◻ ● 150 mg/1mL ◻ do not know

Community pharmacy: Department/County: _ _ ____________________ [free text]

If your pharmacy is not listed, please check the box ◻

Home Hemophilia Treatment Centre: ____________________ [drop-down menu]

What are the main motivations for community pharmacy dispensing?

◻ Proximity to my place of residence ◻ Proximity to my place of work / study or activity

◻ Availability of my community pharmacist

◻ Pre-existing relationship with the hospital pharmacist

◻ Traffic conditions to the hospital ◻ Hospital parking conditions

◻ Opening hours of my community pharmacy ◻ Opening hours of my hospital pharmacy

◻ Other: ……………………………………………………………

Have you ever gone to the community pharmacy to pick up your medication when it is not yet available? ◻ Each time ◻ Often ◻ Sometimes ◻ Never

How do you interact with your community pharmacist?

◻ Face to face ◻ Email ◻ Phone ◻ SMS ◻ Other: …………

**About the emicizumab (Hemlibra®) injection**

About the injection:

◻ I inject myself with the treatment ◻ You inject the patient with the treatment

◻ A relative injects me with the treatment ◻ A nurse injects the treatment

Inject the entire volume of the vial(s)? ◻ yes ◻ no ◻ do not know

Do you encounter any practical difficulties in injecting? ◻ yes ◻ no

- If yes, which: ◻ Placement of the transfer needle ◻ Transfer of the drug into the syringe

◻ During the administration ◻ Waste management

Do you consider the administration kit to be suitable?

◻ yes ◻ no If no, why? :____________

Keeping your hemophilia health record is:

◻ systematic ◻ approximately ◻ not held ◻ do not know

Do you use an health record/electronic hemophilia app?:

◻ yes ◻ no If yes, which:____________

**About specific situations**

Do you know what to do if you miss a dose? ◻ yes ◻ no

- How common is this situation? ◻ Each time ◻ Often ◻ Sometimes ◻ Never
- What do you have in place to prevent missed doses?

◻ Calendar ◻ Alarm ◻ Third party (doctor, pharmacist, nurse) ◻ Other

Do you ever forget to refill your prescription? ◻ Each time ◻ Often ◻ Sometimes ◻ Never

Have you ever reported an adverse reaction to emicizumab (Hemlibra®)? ◻ yes ◻ no

Have you informed the hospital pharmacist in the event of a bleeding situation?

◻ yes: What did he/she advise you to do: ◻ Call the Hemophilia Treatment Centre ◻ Call emergency services ◻ Call the attending physician ◻ Other: ………………………………

◻ no : What did you do? ◻ Use of FVIII according to medical prescription

◻ Use of a bypass agent (NovoSeven® or FEIBA®) according to medical prescription

◻ Other: ………………………………………….

**About your relationship with the Hemophilia Treatment Centre**

What is your overall level of satisfaction with the information provided by your Hemophilia Treatment Centre team about your emicizumab (Hemlibra®) medication?

◻ Very satisfied ◻ Somewhat satisfied ◻ Somewhat not satisfied ◻ Not at all satisfied

What is your overall level of satisfaction with the organization of the transition to the community pharmacy?

◻ Very satisfied ◻ Somewhat satisfied ◻ Somewhat not satisfied ◻ Not at all satisfied

**About your relationship with the community pharmacist**

In your community pharmacy, the staff who dispense emicizumab (Hemlibra®) are usually:

◻ Pharmacist ◻ Preparator ◻ Student pharmacist ◻ do not know

If necessary, is there a confidentiality area where you can talk with your pharmacist?

◻ yes ◻ no ◻ Other, specify: ……………………………………

What is your overall level of satisfaction with:

- Advice from the community pharmacist regarding your treatment?

◻ Completely satisfied ◻ Somewhat satisfied ◻ Rather not satisfied ◻ Not at all satisfied ◻ Not applicable

Any advice from the community pharmacist regarding your prescription refill arrangements?

◻ Completely satisfied ◻ Somewhat satisfied ◻ Rather not satisfied ◻ Not at all satisfied ◻ Not applicable

Advice from the community pharmacist on the use of the injection kit?

◻ Completely satisfied ◻ Somewhat satisfied ◻ Rather not satisfied ◻ Not at all satisfied ◻ Not applicable

- Your relationship with the community pharmacist?

◻ Completely satisfied ◻ Somewhat satisfied ◻ Rather not satisfied ◻ Not at all satisfied

**About your daily life**

What transportation do you use to get to your community pharmacy?

◻ By foot ◻ By car ◻ By public transport ◻ Ambulance ◻ Other transportation: ____________

In general, you go to the community pharmacy from:

◻ Where you live ◻ Your place of work, study or activity ◻ Other: _____________

What is the estimated door-to-door travel time to your community pharmacy:

- from your place of residence: _ _ _ minutes
- from your place of work, study or activity: _ _ _ minutes
- from another location? _ _ _ minutes
- Time spent on site? _ _ _ minutes

Does this travel time seem to you ...?

◻ Completely satisfied ◻ Somewhat satisfied ◻ Rather not satisfied ◻ Not at all satisfied

When do you choose to pick up your medication?

◻ On weekdays ◻ Saturday morning if open

◻ Before 10 a.m. ◻ between 10 a.m. and 4 p.m. ◻ after 4 p.m.

**Concerning the conditions of accessibility to the community pharmacy**

Please enter the postal addresses below. Coordinates are not retained after calculation. Only travel times and distances will be analyzed. Your data will remain irreversibly anonymous.

☞ **Survey 123 geographic module to measure actual travel times:**

**- community pharmacy location - residence**

**- location of community pharmacy - location of work**

**About your satisfaction level**

What is your overall level of satisfaction with the dispensing of your treatment in the community pharmacy?

◻ Completely satisfied ◻ Somewhat satisfied ◻ Rather not satisfied ◻ Not at all satisfied

**Comment: ………………………………………………………………………………………………**

**……………………………………………………………………………………………………………**

Thank you for your participation
